# A general strategy for developing cell-permeable photo-modulatable organic fluorescent probes for live-cell super-resolution imaging

**DOI:** 10.1038/ncomms6573

**Published:** 2014-11-20

**Authors:** Deng Pan, Zhe Hu, Fengwu Qiu, Zhen-Li Huang, Yilong Ma, Yina Wang, Lingsong Qin, Zhihong Zhang, Shaoqun Zeng, Yu-Hui Zhang

**Affiliations:** 1 Britton Chance Center for Biomedical Photonics, Wuhan National Laboratory for Optoelectronics-Huazhong University of Science and Technology, Wuhan 430074, China; 2 Key Laboratory of Biomedical Photonics of Ministry of Education, Department of Biomedical Engineering, Huazhong University of Science and Technology, Wuhan 430074, China

## Abstract

Single-molecule localization microscopy (SMLM) achieves super-resolution imaging beyond the diffraction limit but critically relies on the use of photo-modulatable fluorescent probes. Here we report a general strategy for constructing cell-permeable photo-modulatable organic fluorescent probes for live-cell SMLM by exploiting the remarkable cytosolic delivery ability of a cell-penetrating peptide (rR)_3_R_2_. We develop photo-modulatable organic fluorescent probes consisting of a (rR)_3_R_2_ peptide coupled to a cell-impermeable organic fluorophore and a recognition unit. Our results indicate that these organic probes are not only cell permeable but can also specifically and directly label endogenous targeted proteins. Using the probes, we obtain super-resolution images of lysosomes and endogenous F-actin under physiological conditions. We resolve the dynamics of F-actin with 10 s temporal resolution in live cells and discern fine F-actin structures with diameters of ~80 nm. These results open up new avenues in the design of fluorescent probes for live-cell super-resolution imaging.

## Abstract (web summary)

Single-molecule localization microscopy depends on the use of photo-modulatable fluorescent probes; however, many cannot be used in live-cell studies due to poor cell permeability. Pan *et al.* present a strategy for constructing cell-permeable probes and use it to image actin filament dynamics and lysosomes.

## Graphical abstract

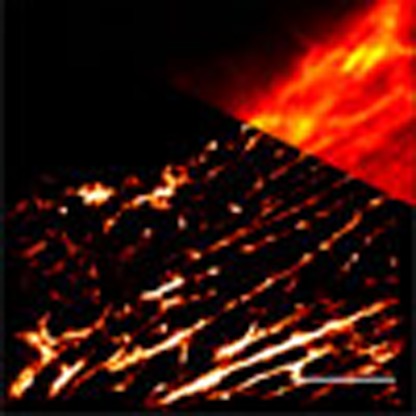

Recent developments in single-molecule localization microscopy (SMLM), such as photo-activated localization microscopy^1^,^2^ and stochastic optical reconstruction microscopy^3^, have enabled biological structures to be defined with a spatial resolution beyond the diffraction limit, providing powerful techniques to obtain exciting new insight into the nanoscale structures and dynamics of biological samples^4^,^5^. In contrast to deterministic far-field super-resolution imaging methods, such as stimulated emission depletion^6^ and structured illumination microscopy^7^, SMLM does not require sophisticated microscopes but instead critically relies on the use of photo-activatable, photo-convertible or photo-switchable (termed photo-modulatable) fluorescent probes^8^. Organic fluorophore-conjugated antibodies used in immunofluorescence techniques have been well-established. However, these probes are primarily restricted to fixed cells^5^,^9^. To specifically label intracellular proteins in intact cells for live-cell SMLM, several strategies have been developed, each with their own limitations. One strategy exploits fluorescent proteins (FPs) because they are live-cell compatible. However, the generally low-fluorescence quantum yield and poor photostability of FPs have resulted in only a few suitable photo-modulatable FPs (for example, photo-activatable green fluorescent protein (PAGFP), PAmCherry, PAtagRFP and tdEosFP) being used successfully in live-cell SMLM so far^10^. Moreover, the overexpression of these FPs may lead to artifacts, such as protein aggregation or inappropriate localization due to saturation of the protein targeting machinery. FP (diameter 3–4 nm) fusions also substantially increase the size of the protein and can interfere with biological activity^11^. The second strategy relies on the combination of a genetically encoded target protein (or peptide) with a separate synthetic probe consisting of a photo-modulatable organic fluorophore and a recognition unit, such as SNAP-tags^12^, TMP-tags^13^ or Halo-tags^14^. However, for this strategy, both the organic fluorophore and the recognition unit of the synthetic probe must be cell permeable for live-cell imaging, which severely restricts the number of the synthetic probes. Moreover, most of photo-modulatable organic fluorophores (for example, photo-caged fluorophores and Alexa 647) with excellent optical properties cannot be used in this strategy due to their poor cell permeability, particularly after conjugation with a recognition unit^15^. Furthermore, the large protein tags (for example, SNAP-Tag 20 kDa, eDHFR/TMP-Tag 18 kDa and Halo-Tag 35 kDa) used in these methods can sterically interfere with protein function. Overexpressed fusion proteins rather than endogenous proteins are labelled in these procedures, which may also cause overexpression artifacts.

Here we develop a new general strategy for constructing cell-permeable photo-modulatable organic fluorescent probes for live-cell super-resolution imaging by utilizing the remarkable cytosolic delivery ability of a cell-penetrating peptide (CPP), (rR)_3_R_2_ (r: D-Arg, R: L-Arg). CPPs are short peptides that can penetrate the cell membrane and translocate linked cell-impermeable cargoes into live cells. Although they are able to enter live cells efficiently, most CPPs are often trapped within punctate vesicles and have difficulty being released to the cytosol to exert their functions inside cells^16^. We have recently developed a short CPP (rR)_3_R_2_ that can efficiently deliver small membrane-impermeable molecules into the cytosol rather than punctate vesicles in live cells^17^. Based on the excellent properties of (rR)_3_R_2_, including low cytotoxicity, ease of synthesis, high uptake efficiency and efficient cytosolic delivery^17^, we designed novel photo-modulatable organic fluorescent probes consisting of the CPP (rR)_3_R_2_, a organic fluorophore (cell impermeable) and a recognition unit (cell impermeable). Our results indicate that these organic probes are not only cell permeable but can also specifically and directly label endogenous proteins. Using these probes, we obtain super-resolution images of F-actin and lysosomes in live cells. Remarkably, we monitor the dynamics of F-actin under physiological conditions with 10 s temporal resolution. To the best of our knowledge, the probes presented here are the first cell-permeable photo-modulatable organic fluorescent probes that can directly label intracellular endogenous targeted proteins in live cells for super-resolution imaging. Considering that the organic fluorophore and the recognition unit of the probes can be easily replaced with other organic dyes and recognition groups, respectively, which do not need to be cell permeable anymore, thus greatly expanding the number of organic fluorophores and recognition groups that can be used inside live cells for SMLM.

## Results

### Design and synthesis of the organic fluorescent probes

**Figure 1:**
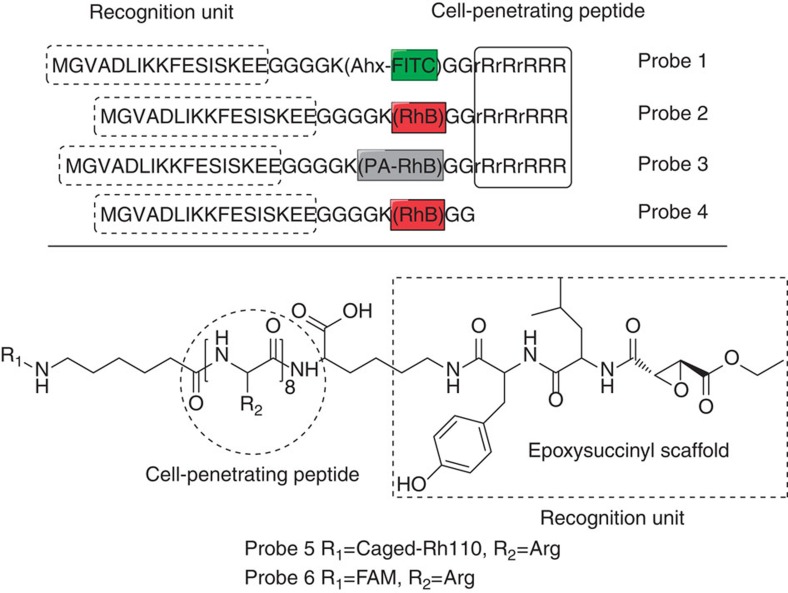
Structures of constructed probes 1–6. Ahx, ε-aminocaproic acid; FITC, fluorescein isothiocyanate; RhB, rhodamine B; PA-RhB, photoactivatable rhodamine B derivative; Caged-Rh110, caged rhodamine 110 derivative; FAM, 5(6)-carboxyfluorescein; r, D-Arginine.

We designed and synthesized the organic fluorescent probes 1–6 (Fig. 1, Supplementary Fig. 1) using standard solid-phase peptide synthesis^18^,^19^,^20^. The probes 1–3 have the following four components: (a) Lifeact (MGVADLIKKFESISKEE), a 17-amino-acid peptide, which can specifically and reversibly bind to the F-actin cytoskeleton without compromising cellular processes^21^; (b) an (rR)_3_R_2_ peptide to engender cellular permeability of the probe^17^; (c) six glycine residues as a spacer and (d) a fluorophore (fluorescein isothiocyanate (FITC), rhodamine B (RhB) or a photo-activatable rhodamine B derivative (PA-RhB) that has a high fluorescence quantum yield, high photochemical stability and a large contrast ratio between the fluorescent and the dark state^22^). We also generated probe 4, which does not contain a CPP, as a control of probe 2 (Fig. 1). Compared with probe 3, probes 5 and 6 contain a different recognition unit, an epoxysuccinyl scaffold, which can selectively form covalent bonds with cysteine cathepsins (typically localized in lysosomes)^19^,^20^ and a different fluorophore (a caged rhodamine 110 derivative (Caged-Rh110) or 5(6)-carboxyfluorescein (FAM))^23^.

### Effect of (rR)_3_R_2_ on the labelling of the probes for F-actin

**Figure 2:**
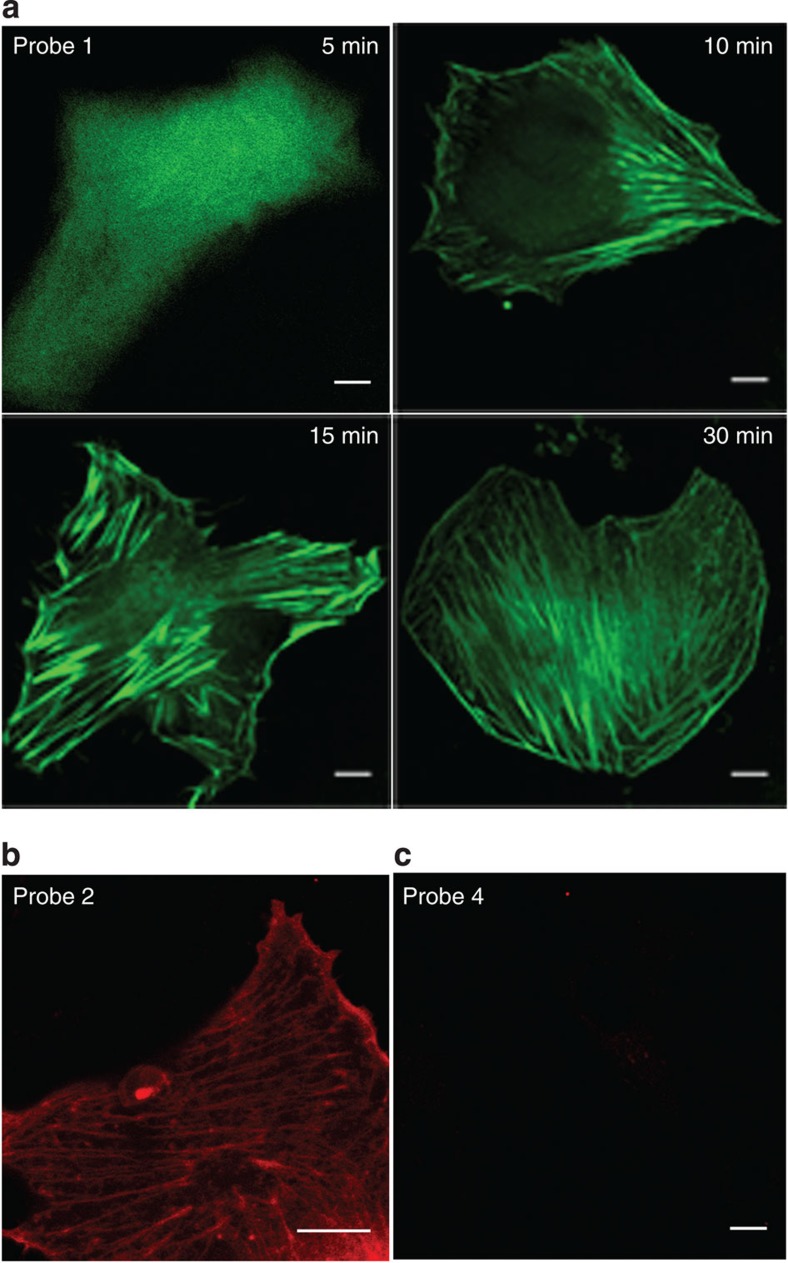
The intracellular distribution of probes 1, 2 and 4 in live BSC-1 cells. Confocal microscopy images of live BSC-1 cells after incubation with (**a**) FITC-labelled probe 1 (15 μM) for 5, 10, 15 and 30 min, (**b**) RhB-labelled probe 2 (15 μM) for 30 min and (**c**) probe 4 (15 μM) for 30 min. Scale bars, 10 μm.

Previous studies showed that the intracellular distribution (for example, diffuse cytosolic distribution or entrapment in punctate vesicles) of CPPs was affected by their cargoes^24^. Therefore, we first determined whether the short peptide (rR)_3_R_2_ can efficiently transport the recognition peptide (Lifeact) and the dye (FITC or RhB) of probes 1 and 2 into live cells and more importantly into the cytosol rather than punctate vesicles to access F-actin. Trypan blue was used to exclude the dead cells and quench the extracellular fluorescence from the probes bound to either the cell membrane or the dish surface^25^. Figure 2a indicates that after incubation with BSC-1 cells, FITC-labelled probe 1 entered the live cells efficiently within 5 min and underwent diffuse localization throughout the cytoplasm. Few punctate signals were observed in Fig. 2 during the time course from 5 to 30 min, suggesting that probe 1 did not enter the cells via endocytosis, which is in good agreement with our previous report^17^. By contrast, no obvious fluorescent signal was detected for probe 4, which did not contain a CPP (Fig. 2c, Supplementary Fig. 2). These results suggest that the CPP (rR)_3_R_2_ can efficiently deliver the cell-impermeable recognition unit and the dye into the cytosol of live cells. Over time, probe 1 was gradually bound to the filamentous structures (Fig. 2a). After 30 min, both FITC-labelled probe 1 and RhB-labelled probe 2 stained the filamentous structures efficiently (Fig. 2a,b), suggesting that altering the fluorophore attached to the probe does not influence its labelling. The staining of PA-RhB-labelled probe 3 was not determined because PA-RhB is difficult to detect with a confocal microscope.

**Figure 3:**
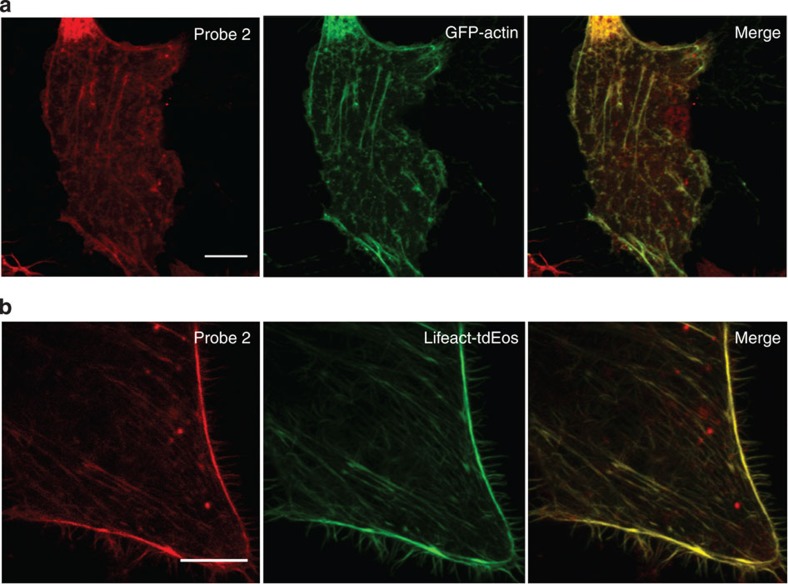
Co-localization studies employing GFP–actin or Lifeact-tdEosFP as the standard actin markers. Live BSC-1 cells transiently transfected with (**a**) GFP–actin (green) or (**b**) Lifeact-tdEosFP (green) were stained with RhB-labelled probe 2 (red, 15 μM) for 30 min and imaged by confocal microscopy. Scale bars, 10 μm.

Next, we investigated the specificity of labelling F-actin with the probes by performing co-localization studies employing both GFP-actin^26^ and Lifeact-tdEosFP^27^ as the standard actin markers. The results indicate that RhB-labelled probe 2 co-localized well with the GFP–actin in BSC-1 cells (Fig. 3a, Pearson’s coefficient: 0.837), HeLa cells (Pearson’s coefficient: 0.878) and NIH/3T3 cells (Pearson’s coefficient: 0.853; Supplementary Fig. 3), suggesting that the conjugation with the polycationic CPP (rR)_3_R_2_ has little effect on the high labelling specificity of the recognition unit Lifeact towards actin. These results were further confirmed by the excellent co-localization of RhB-labelled probe 2 with Lifeact-tdEosFP (Fig. 3b, Pearson’s coefficient: 0.939). There were some off-target punctate signals detected in Fig. 3, possibly arising from the probes bound to the cell membrane because a careful wash before imaging could decrease the punctate signals.

### Super-resolution imaging of F-actin in live cells

Based on the excellent cell permeability and high specificity of probes 1 and 2, we investigated the utility of probes 1–3 in a live-cell super-resolution microscopy experiment. After incubation with live BSC-1 cells, no photoblinking was observed for FITC-labelled probe 1 (Supplementary Movie 1). Therefore, although FITC has been used for SMLM in fixed cells^28^, it is not suitable for live-cell super-resolution imaging without additives most likely because the endogenous thiols in the live cells, which provide triplet state depletion of FITC, were not sufficient for reversible photoswitching. For RhB-labelled probe 2, although the photoblinking was observed (Supplementary Movie 2), it was difficult to construct a super-resolution image due to the low signal-to-noise ratio of RhB.

The non-fluorescent PA-RhB fluorophore has been reported to be switched on by ultraviolet light, to be thermally reverted to a dark state, and to be excited by 560-nm laser light, emitting optimally at 600 nm in fixed cells^22^. However, it is unknown whether the live-cell environment changes the properties of PA-RhB and whether the PA-RhB fluorophore can be used for live-cell super-resolution imaging. After incubation with live BSC-1 cells for 30 min, PA-RhB-labelled probe 3 was observed to be blinking inside of the cells when irradiated with 561-nm laser light (0.025–0.33 kW cm^−2^) without any ultraviolet light activation (Supplementary Movie 3). Moreover, no obvious photobleaching of probe 3 occurred within at least 30 min, even at the maximum intensity (0.33 kW cm^−2^) of 561-nm laser radiation without additional photoactivation (Supplementary Fig. 4). By contrast, in BSC-1 cells, obvious photobleaching was observed for Lifeact-tdEosFP after 4 min (~8,000 frames) at an excitation power of 0.165 kW cm^−2^ (561 nm) with an ultraviolet light activation (405 nm) (Supplementary Fig. 4). The PA-RhB fluorophore has been shown to be spontaneously activated without photo-induced activation for densely labelled samples^22^. Because probe 3 is a small-molecule organic probe, it can bind to the target protein with little steric hindrance, which may result in high density labelling of actin; this high density labelling most likely led to obvious spontaneous activation. By contrast, in fixed cells, only the first 100–1,000 frames could be visualized for the PA-RhB-labelled tubulin antibody without photoactivation using the limited spontaneously activated molecules^22^ possibly because the labelling density^29^ of the antibody is much lower than that of the small-molecule organic probe 3. Previous reports claimed that the low affinity of the recognition peptide Lifeact for F-actin in live cells allowed for the replenishment of bleached fluorophores and led to rapid fluorescence recovery of the probe after photobleaching^21^,^30^. However, the photobleaching of Lifeact-tdEosFP after 4 min was still obvious, whereas for PA-RhB-labelled probe 3, the photobleaching was not observed even at 30 min, suggesting that the anti-photobleaching characteristic of probe 3 is most likely due to both the excellent photostability of the PA-RhB fluorophore and the low affinity of Lifeact for F-actin.

**Figure 4:**
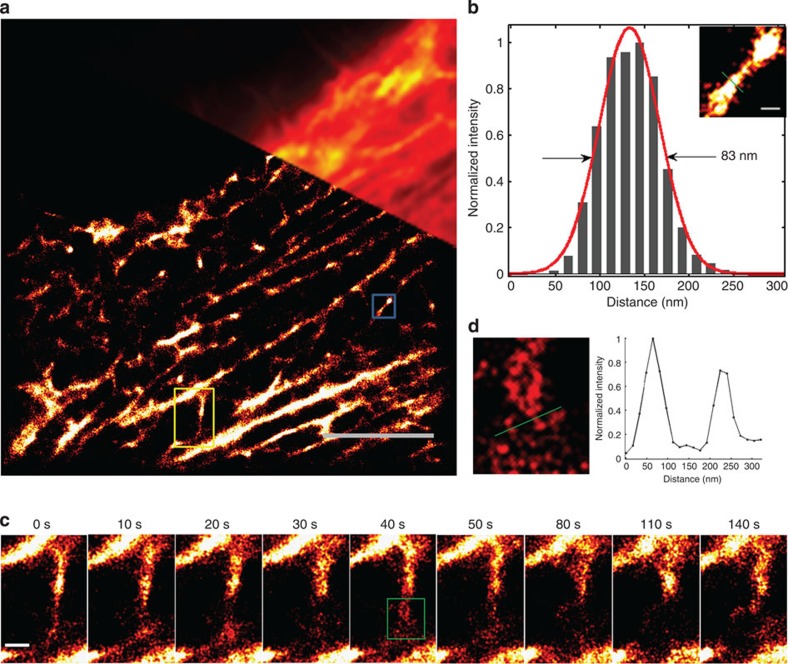
Reconstructed images of actin filaments in a live BSC-1 cell stained with PA-RhB-labelled probe 3. (**a**) Diffraction-limited TIRF (upper right) and super-resolution (lower left) images. (**b**) Enlarged super-resolution image of the blue-boxed region in **a** and cross-sectional profiles of the actin filaments with full-width at half-maximum (FWHM) of 83 nm. (**c**) Enlarged super-resolution time-lapse images of the yellow-boxed region in **a**. Each image was reconstructed from 1,000 frames at 100 Hz. (**d**) Enlarged image of the boxed region in **c** at 40 s. Scale bars, (**a**) 5 μm, (**b**) 200 nm and (**c**) 500 nm.

To determine the best imaging conditions for probe 3 in live cells, we first evaluated the effect of the excitation power. The results indicate that increasing the excitation power (561 nm) from 6 mW (0.025 kW cm^−2^) to 40 mW (0.165 kW cm^−2^) led to an obvious improvement of signal intensity (Supplementary Fig. 5). No significant differences were observed between the images acquired at 40 mW (0.165 kW cm^−2^) or 80 mW (0.33 kW cm^−2^; Supplementary Fig. 5). Considering that higher excitation powers cause more photodamage, we performed our following experiments with an excitation power of 40 mW. Second, we acquired images at different frame acquisition rates (100, 83, 66 and 58 Hz). The results show that slower frame rates yielded higher signal densities (Supplementary Fig. 5). When reconstructing the SMLM images with the Parallel Localization of Multiple Emitters via Bayesian information criterion Recommendation (PALMER) algorithm for high density localization^31^, we found that the signals at 83, 66 and 58 Hz were too dense to be accurately localized. We therefore acquired images consistently at 100 Hz with an excitation power of 0.165 kW cm^−2^ (561 nm). Then we reconstructed SMLM images with 200, 500, 1,000 and 2,000 frames. The results indicate that 1,000 frames (10 s temporal resolution) were sufficient to obtain a smooth and continuous super-resolution image of actin filaments (Fig. 4, Supplementary Fig. 6). Compared with the diffraction-limited image, a much higher spatial resolution was obtained (Fig. 4a), and remarkably the fine F-actin structures with diameters of ~80 nm could be discerned (Fig. 4b). These results indicate that our PA-RhB-labelled probe 3 can be used for super-resolution imaging of endogenous actin in live cells with high spatiotemporal resolution under physiological conditions without needing any additive or additional photoactivation.

### New insight into the dynamic behaviours of F-actin

F-actin plays key roles in many important cellular processes such as regulation, mechanosensation, vesicle and organelle movement, cell motility and cell division^32^,^33^. Filaments in the network are relatively unstable, undergoing continuous changes, such as polymerization and depolymerization, bending and twisting, and severing and branching, enabling the network to remodel itself in response to external or internal stimuli^34^. However, the diameter of F-actin is far <100 nm and therefore difficult to observe using ordinary light microscopy.

Based on the excellent optical properties of PA-RhB-labelled probe 3, we tried to monitor the dynamic behaviours of F-actin in live cells. We acquired 15,000 images at 100 Hz with a total acquisition time of 2.5 min (excitation power: 0.165 kW cm^−2^ (561 nm)). By rendering images from sub-stacks of 1,000 frames, a series of time-lapse super-resolution images were obtained (Fig. 4c) at a time-series interval of 10 s. Although most actin filaments remained still during the acquisition time, remarkably, these time-lapse super-resolution images reveal a dynamic procedure in which an actin bundle rearranged its connection to the network (Fig. 4c). The following is one possible interpretation of the dynamic procedure. The actin bundle was severed (20 s), then formed a loose twist structure made from two filaments (40 s, Fig. 4d), and finally extended the severed actin bundle establishing a new connection with another adjacent F-actin (140 s). To the best of our knowledge, this is the first report of the dynamics of F-actin in live cells with nanoscale spatial resolution and 10 s temporal resolution. We also recorded time-lapse super-resolution images of actin dynamics in filopodia in live cells (Supplementary Movie 4).

### Super-resolution imaging of lysosomes in live cells

To evaluate the generality and expanded utility of our strategy, we also constructed another organic fluorescent probe 5 (Fig. 1), which contains a different recognition unit and a fluorophore from probe 3. The recognition unit of probe 5 is an epoxysuccinyl scaffold, which can selectively form covalent bonds with cysteine cathepsins (typically localized in lysosomes)^19^,^20^. We used Caged-Rh110 instead of PA-RhB in probe 5 because Caged-Rh110 is stable under acidic conditions^23^ (pH≈5 in lysosomes), while PA-RhB is pH sensitive^22^. The non-fluorescent Caged-Rh110 can be uncaged with a 405-nm laser light and excited at 473 nm, emitting optimally at 520 nm^23^.

**Figure 5:**
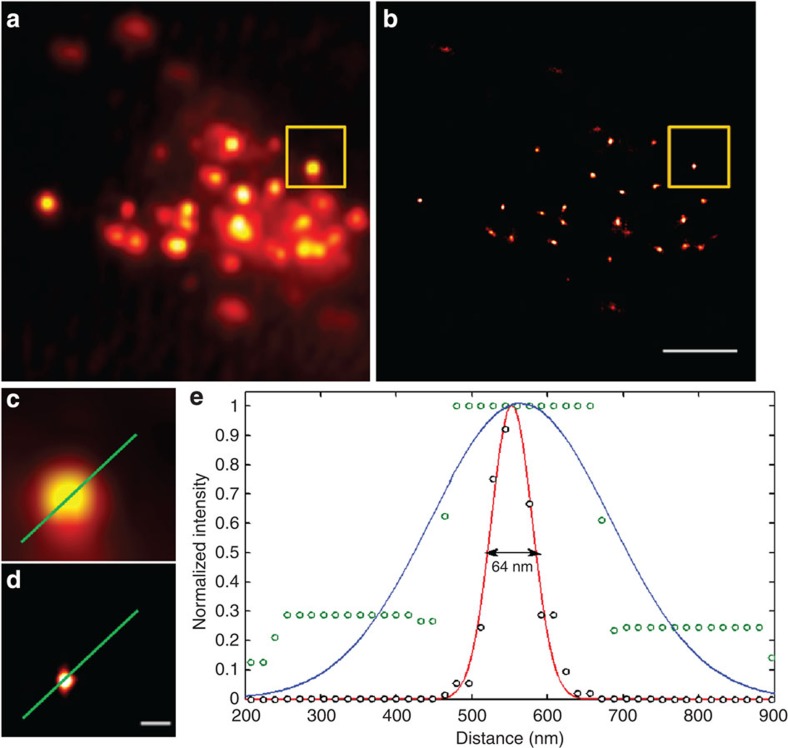
Reconstructed images of lysosomes in a live HeLa cell stained with Caged-Rh110-labelled probe 5. (**a**) Diffraction-limited TIRF. (**b**) Super-resolution image. (**c**) Enlarged TIRF image of the boxed region in **a**. (**d**) Enlarged super-resolution image of the boxed region in **b**. Super-resolution image was reconstructed from 500 frames at 260 Hz. (**e**) Cross-sectional profiles of lysosome shown in **c**,**d** (diffraction-limited, blue line; super-resolved, red line with a full-width at half-maximum (FWHM) of 64 nm). Scale bars, (**b**) 2 μm and (**d**) 200 nm.

Previous reports have shown that the probes containing the epoxysuccinyl scaffold can specifically label lysosomes^19^,^20^,^35^, which was further confirmed by the excellent co-localization of LysoTracker Red with probe 6 (Pearson’s coefficient: 0.850), a control of probe 5, which contains a FAM for observing with an ordinary light microscopy (Supplementary Fig. 7). To obtain the super-resolution images of lysosomes, live HeLa cells stained with probe 5 were firstly exposed to a high level of 473 nm light (0.38 kW cm^−2^) to photobleach any previously uncaged molecules. Then, images were acquired under continuous illumination at 473 nm (0.38 kW cm^−2^) at a frame rate of 260 Hz with an ultraviolet light intermittent activation (405 nm). We reconstructed super-resolution images of lysosomes from 500 frames (a total acquisition time of 3.8 s) using the PALMER algorithm^31^ and obtained a super-resolution image of lysosomes with a full-width at half-maximum of 64 nm (Fig. 5). A movie of the live-cell imaging of lysosomes stained with probe 5 was shown in Supplementary Movie 5. These results demonstrate that Caged-Rh110-labelled probe 5 can be used to image lysosomes with a resolution beyond optical diffraction limit in live cells.

## Discussion

Organic photo-modulatable fluorophores offer several important photophysical advantages over FPs, including smaller sizes, broader spectral coverage, higher fluorescence quantum yields and better photostabilities; therefore, use of organic fluorophores could potentially improve the spatial and temporal resolution of SMLM^15^. Although various photo-modulatable organic fluorophores with excellent optical properties have been reported^9^, necessary cell permeability, particularly after conjugation with a recognition unit, severely restricts the application of these organic fluorophores in live-cell SMLM. To address these problems, the general strategy in previous reports has involved modifying the structures of organic fluorophores or the recognition unit chemically to improve their permeability, which is expensive and time consuming and, in particular, might eliminate the photo-modulatable properties of organic fluorophores or decrease the affinity of the recognition unit. Here we develop a new general strategy for constructing cell-permeable photo-modulatable organic fluorescent probes for live-cell super-resolution imaging by exploiting the remarkable cytosolic delivery ability of (rR)_3_R_2_. We construct two novel cell-permeable photo-modulatable organic fluorescent probes, PA-RhB-labelled probe 3 and Caged-Rh110-labed probe 5, which contain different recognition units and fluorophores. Our results indicate that both of the probes can enter live cells efficiently and directly label endogenous targeted proteins for super-resolution imaging. Using the probes, we obtain super-resolution images of lysosomes (~ 60 nm) and endogenous F-actin (~ 80 nm) under physiological conditions.

Remarkably, PA-RhB-labelled probe 3 can be excited by a single laser light under physiological conditions without other additives, such as redox chemicals required for most other organic probes^28^ or additional photoactivation, thereby greatly reducing the complexity of the experiment and the phototoxicity caused by the second ultraviolet light activation. Moreover, PA-RhB-labelled probe 3 has an excellent anti-photobleaching property, making it potentially very valuable for long-term real-time super-resolution imaging.

Resolving the fast dynamics of actin networks requires both high spatial resolution and temporal resolution. Although the photo-modulatable FP Lifeact-tdEosFP has been used successfully for live-cell SMLM, the highest temporal resolution that can be achieved by Lifeact-tdEosFP was 50 s (ref. 30). Applying higher activation laser intensities leads to higher emitter densities, which can improve the temporal resolution; however, this application also results in the quick photobleaching of Lifeact-tdEosFP. Alternatively, using PA-RhB-labelled probe 3, we resolve the dynamics of F-actin with 10 s temporal resolution in live cells, and the fine F-actin structures with diameters of ~80 nm could be discerned, thereby allowing us to follow the dynamics with high spatiotemporal resolution. Our results reveal a connection rearrangement procedure of an actin bundle, which has never been previously reported in living cells, therefore providing new insights into the dynamic behaviours of F-actin *in vivo*.

Taken together, we anticipate that probes 3 and 5 will be a useful tool for super-resolution imaging and may substantially benefit ultrastructural characterizations of live cells. Notably, the easy replacement of the organic fluorophore and the recognition unit of probes 3 and 5 with other cell-impermeable organic dyes and recognition groups (for example, peptides and protein substrates) will greatly expand the number of organic dyes and recognition groups that can be used inside live cells for SMLM. The results here provide a new general strategy for developing cell-permeable organic fluorescent probes for live-cell super-resolution imaging. Studies using other dyes (for example, Alexa 647 and Cy3B) and recognition units (for example, phalloidin^36^, docetaxel^37^, SNAP-tag^12^ and TMP-tag^13^) to construct new cell-permeable probes for live-cell SMLM are currently underway.

## Methods

### Materials

Probes 1–6 were prepared by solid-phase peptide synthesis, purified by preparative high performance liquid chromatography to reach a purity >95%, and their appropriate masses were confirmed by electrospray ionization mass spectrometry (Supplementary Fig. 1).

### Cell culture

BSC-1 cells (African green monkey (*Cercopithecus aethiops*) kidney cells), HeLa and NIH/3T3 (Mouse embryonic fibroblast cell line) cells were purchased from Boster Biological Technology Co. Ltd, Wuhan, China. BSC-1 cells were cultured in Minimum Essential Medium (MEM; Gibco BRL, Grand Island, NY, USA). HeLa and NIH/3T3 cells were cultured in Dulbecco’s modified Eagle’s medium (DMEM; Gibco BRL). All media were supplemented with 10% foetal bovine serum (FBS; Gibco BRL) and maintained at 37 °C in a humidified 5% CO_2_ environment.

### Transfection

BSC-1, HeLa and NIH/3T3 cells were grown overnight in 24-well plates at 37 °C in a 5% CO_2_ atmosphere. After reaching ~80% confluence, the GFP–actin plasmid or the Lifeact-tdEosFP plasmid was transfected into the cells using Lipofectamine 2000 (Invitrogen, Grand Island, NY, USA) according to the manufacturer’s instructions. After 24 h, the transfected cells were digested with trypsin (0.25% EDTA) and seeded into cell culture dishes (glass bottom *Φ*15 mm, NEST Biotechnology Co., Ltd., China) at a density of 1.5 × 10^4^ per well. The cells were grown for an additional 12−24 h before incubation with the indicated probe.

### Labelling live cells with the probes

For F-actin imaging, BSC-1 cells were seeded into cell culture dishes at a density of 1.5 × 10^4^ per well in growth medium (MEM supplemented with 10% FBS, 200 μl). After an overnight incubation, the cells were washed with phosphate-buffered saline (PBS, pH 7.4) for three times. A solution of the indicated probe (15 μM, 200 μl) in PBS was then added, and the cells were incubated in a 5% CO_2_ atmosphere at 37 °C for the indicated (5, 10, 15 and 30 min) times as mentioned in the main text. The supernatant was then discarded, and a solution of Trypan blue (200 μl, 1 mg ml^−1^) in PBS was added to quench the extracellular fluorescence of the probes that were bound to either the cell membrane or the dish surface. After 2 min, Trypan blue was removed, the cells were washed gently twice with PBS and immersed in growth medium prior to optical imaging.

For lysosome imaging, HeLa cells were seeded into cell culture dishes at a density of 2.0 × 10^4^ per well in growth medium (DMEM supplemented with 10% FBS, 200 μl). The cells were then grown at 37 °C in a 5% CO_2_ atmosphere overnight. After washing with PBS for three times, the cells were incubated with probe 5 (6 μM, 200 μl) or probe 6 (15 μM, 200 μl) in PBS for 30 min in a 5% CO_2_ atmosphere at 37 °C. The supernatant was discarded and the cells were rinsed with PBS for three times and incubated in growth medium (DMEM supplemented with 10% FBS, 200 μl) for 5 h. To stain lysosomes, the cells were incubated with LysoTracker Red (500 nM, 200 μl) for 30 min before imaging. Then the cells were washed twice gently with PBS and immersed in growth medium prior to optical imaging.

### Confocal laser scanning microscopy

The fluorescence signals were detected using a Fluoview FV1000 confocal laser scanning microscope (Olympus) equipped with a × 60/1.42 numerical aperture oil-immersion objective lens (FITC channel: excitation (EX) 488 nm, emission (EM) 510–530 nm; GFP channel: EX 488 nm, EM 508–532 nm; tdEosFP channel: EX 488 nm, EM 500–522 nm; RhB channel: EX 543 nm, EM 580–630 nm; FAM channel: EX 488 nm, EM 505–540 nm and LysoTracker Red channel: excitation (EX) 543 nm, emission (EM) 560–580 nm). All fluorescence images were analyzed with Image J software.

### Single-molecule localization-based super-resolution imaging

The single-molecule imaging experiments were performed with a laboratory-built total internal reflection fluorescence (TIRF) microscope setup consisting of an Olympus IX 71 inverted optical microscope, a × 100/numerical aperture1.49 oil-immersion TIRF objective (UAPON 100XOTIRF, Olympus), an iXon 897 EMCCD camera (Andor Tech.), a 561-nm diode-pumped solid-state laser (CNILaser, China) for PA-RhB excitation, a 473-nm diode-pumped solid-state laser (CNILaser) for Caged-Rh110 excitation and a 405-nm laser diode (CNILaser) for caged-Rh110 activation. An electronic shutter (UNIBLITZ VS14, Vincent Associates) was used to control the duration of laser irradiance, and a dichroic mirror (Di01-R488/561, Semrock) and long-pass filter (BLP01–561R-25, Semrock) were used to separate the collected fluorescence from the scattering laser and impurity fluorescence^31^. For live-cell F-actin imaging, a wavelength of 561 nm with an intensity of ~0.165 kW cm^−2^ was used to record movies at a frame rate of 100 Hz. The data were acquired with the software that was provided by the manufacturer of the camera. For live-cell lysosome imaging, live HeLa cells stained with probe 5 were firstly exposed to a 473 nm light (0.38 kW cm^−2^) to photobleach any previously uncaged molecules. Images were acquired under continuous illumination at 473 nm (0.38 kW cm^−2^) at a frame rate of 260 Hz with an ultraviolet light intermittent activation (405 nm, 5.5–23 W cm^−2^).

### Data analysis

A fast, high-precision image analysis method for high density localization, termed PALMER, was used to reconstruct the super-resolved images^31^. Subsequent rendered images of F-actin were generated from 1,000 video sequences, with no overlap between two adjacent reconstructed images, that is, sequence 1–1,000, 1,001–2,000, 2,001–3,000 and so on. Super-resolution images of lysosomes were reconstructed from 500 frames.

## Author contributions

Experiments were performed primarily by D.P., Z.H., and F.Q. with regular input and guidance from Y.-H.Z. and Z.-L.H. Y.M. and F.Q. contributed to the synthesis of the probes. D.P., Z.H., Y.W., L.Q. Z.Z. S.Z. and Y.-H.Z. analyzed the data. Experimental strategy was designed by Y.-H.Z. The manuscript was written by Y.-H.Z. and D.P. with discussion and improvements from all authors.

## Additional information

**How to cite this article:** Pan, D. *et al.* A general strategy for developing cell-permeable photo-modulatable organic fluorescent probes for live-cell super-resolution imaging. *Nat. Commun.* 5:5573 doi: 10.1038/ncomms6573 (2014).

## Supplementary Material

Supplementary Information Supplementary Figures 1-7

Supplementary Movie 1 An imaging sequence of live BSC-1 cells treated by FITC-labeled probe 1 (15 μM, 30 min) (500 frames, 100 Hz, excitation power: 488 nm, 0.36 kWcm^-2^). Scale bar: 10 μm.

Supplementary Movie 2 An imaging sequence of live BSC-1 cells treated by RhB-labeled probe 2 (15 μM, 30 min) (500 frames, 100 Hz, excitation power: 561 nm, 0.165 kWcm^-2^). Scale bar: 10 μm.

Supplementary Movie 3 An imaging sequence of live BSC-1 cells treated by PA-RhB-labeled probe 3 (15 μM, 30 min) (500 frames, 100 Hz, excitation power: 561 nm, 0.165 kWcm^-2^). Scale bar: 10 μm.

Supplementary Movie 4 A series of time-lapse super-resolution images of actin dynamics in filopodia in live BSC-1 cells stained with PA-RhB-labeled probe 3 (15 μM, 30 min) at a time-series interval of 10 s. Every image was reconstructed by the PALMER algorithm from 1000 frames (0.165 kWcm^-2^, 100 Hz). Scale bar: 2 μm.

Supplementary Movie 5 An imaging sequence of live HeLa cells treated by Caged-Rh110-labeled probe 5 (6 μM, 30 min) (500 frames, 260 Hz, excitation power: 473 nm, 0.38 kWcm^-2^, activation power: 405 nm). Scale bar: 2 μm.
