# Supplementary material for: A general strategy for developing cell-permeable photo-modulatable organic fluorescent probes for live-cell super-resolution imaging
Source: Nat Commun. 2014 Nov 20;5:5573. doi: 10.1038/ncomms6573 (PMC4263135; doi:10.1038/ncomms6573)
Supplement: Supplementary Information — Supplementary Figures 1-7 [file ncomms6573-s1.pdf]

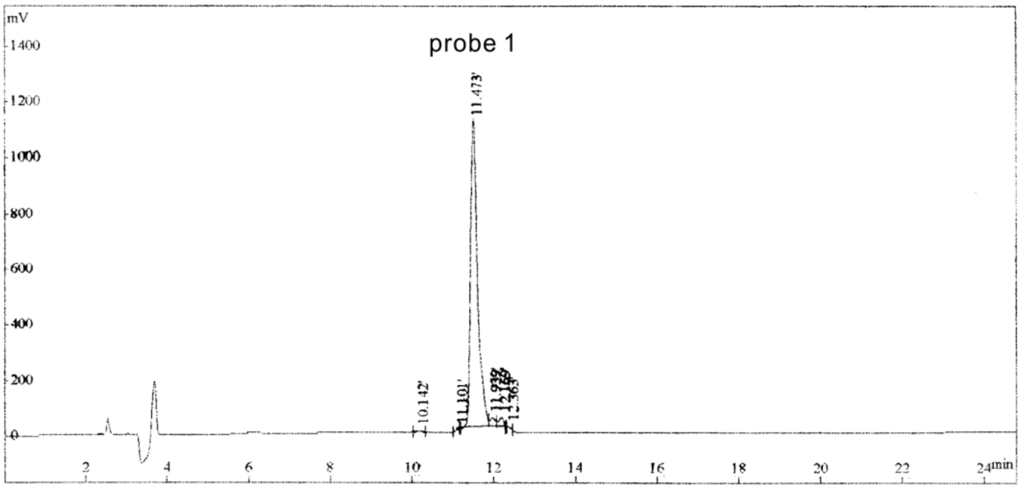

Rank	Time(min)	Conc.(%)	Area	Height
1	10.142	0.3814	52827	6662
2	11.101	0.0723	10008	2223
3	11.473	95.2321	13191066	1109207
4	11.939	2.3183	321112	33202
5	12.169	1.9002	263200	33928
6	12.363	0.0957	13257	3380
Total		100	13851470	1188602

Supplementary Figure 1a. HPLC analysis of probe **1**.

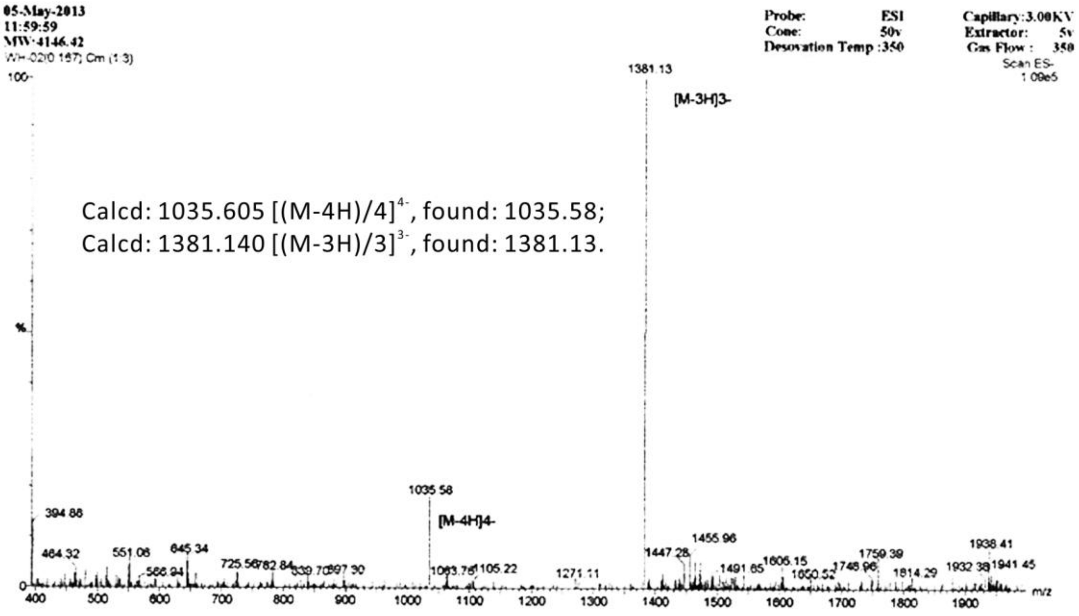

Supplementary Figure 1b. ESI mass spectrum analysis of probe **1**.

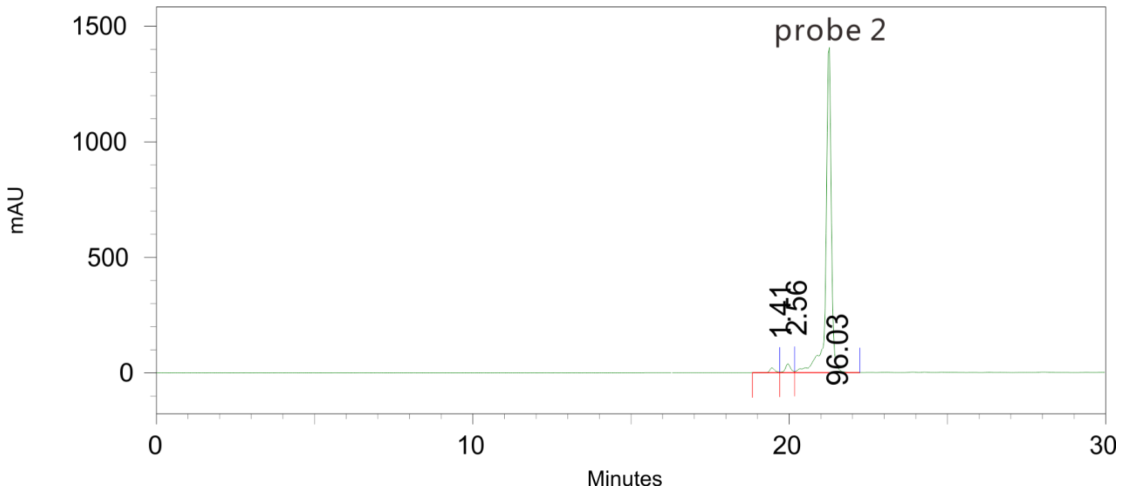

Rank	Time (min)	Conc.(%)
1	19.435	1.41
2	19.940	2.56
3	21.236	96.03
Total		100

Supplementary Figure 1c. HPLC analysis of probe **2**.

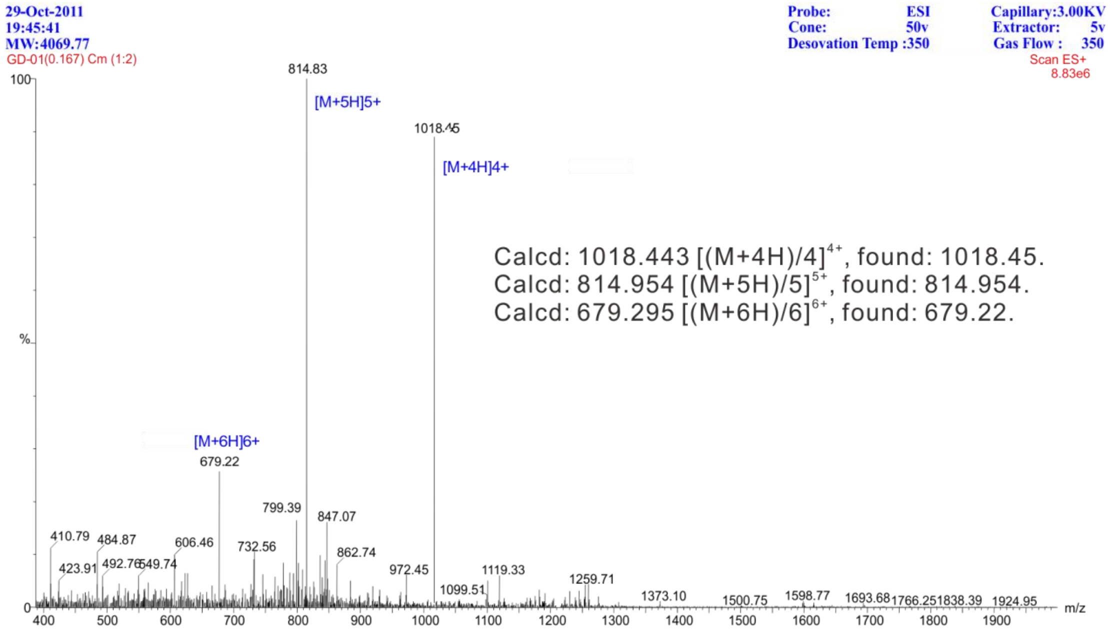

Supplementary Figure 1d. ESI mass spectrum analysis of probe **2**.

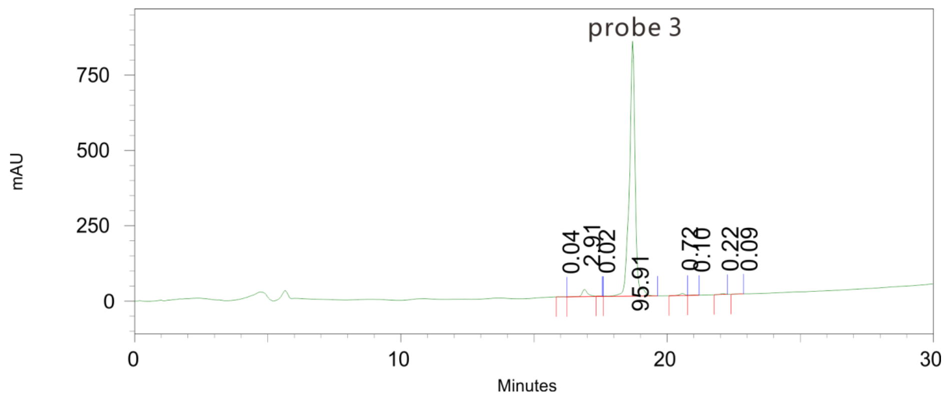

Rank	Time (min)	Conc.(%)
1	16.083	0.04
2	16.877	2.91
3	17.432	0.02
4	18.685	95.91
5	20.554	0.72
6	21.001	0.10
7	22.066	0.22
8	22.767	0.09
Total		100

Supplementary Figure 1e. HPLC analysis of probe **3**.

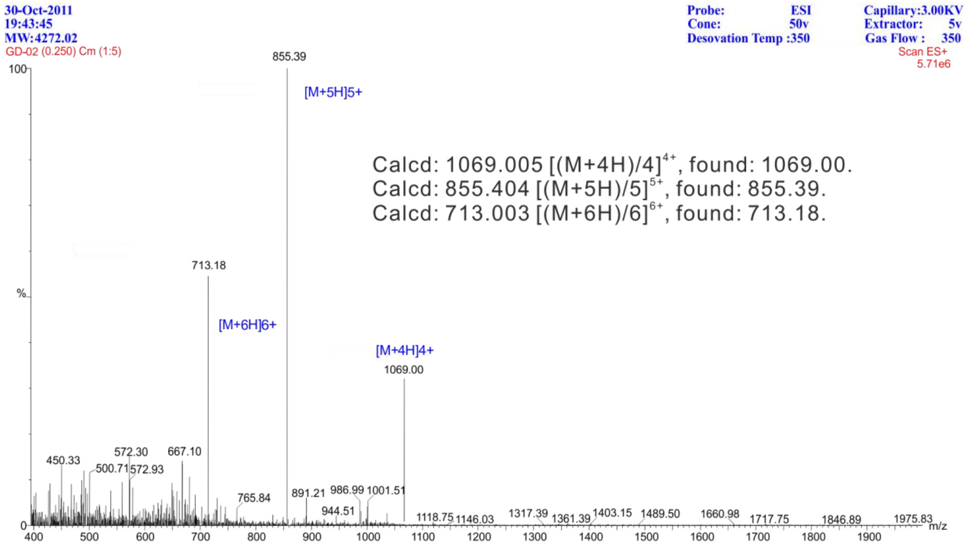

Supplementary Figure 1f. ESI mass spectrum analysis of probe **3**.

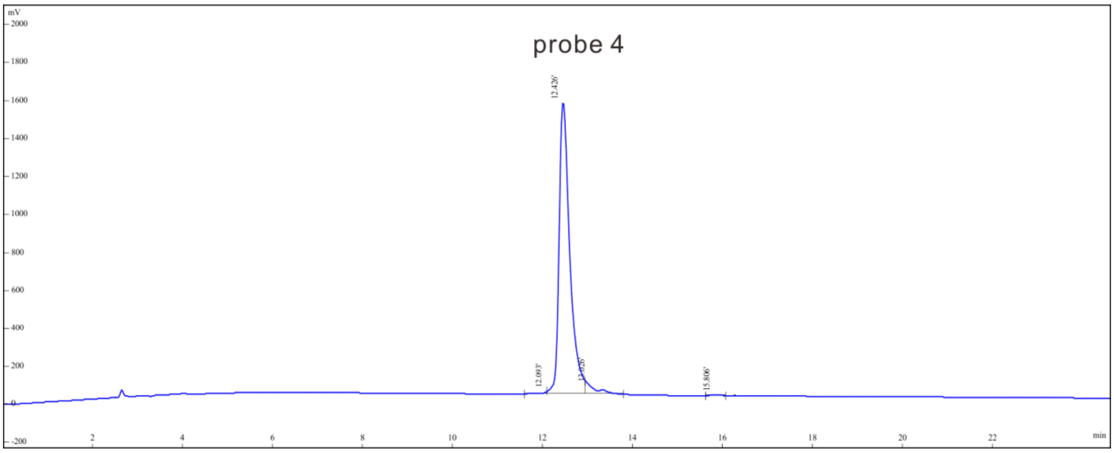

Rank	Time(min)	Conc.(%)	Area	Height
1	12.093	0.4577	121611	16383
2	12.426	95.75	25439547	1542220
3	13.026	3.544	941574	42938
4	15.806	0.2422	64343	4832
Total		100	26567075	1606373

Supplementary Figure 1g. HPLC analysis of probe **4**.

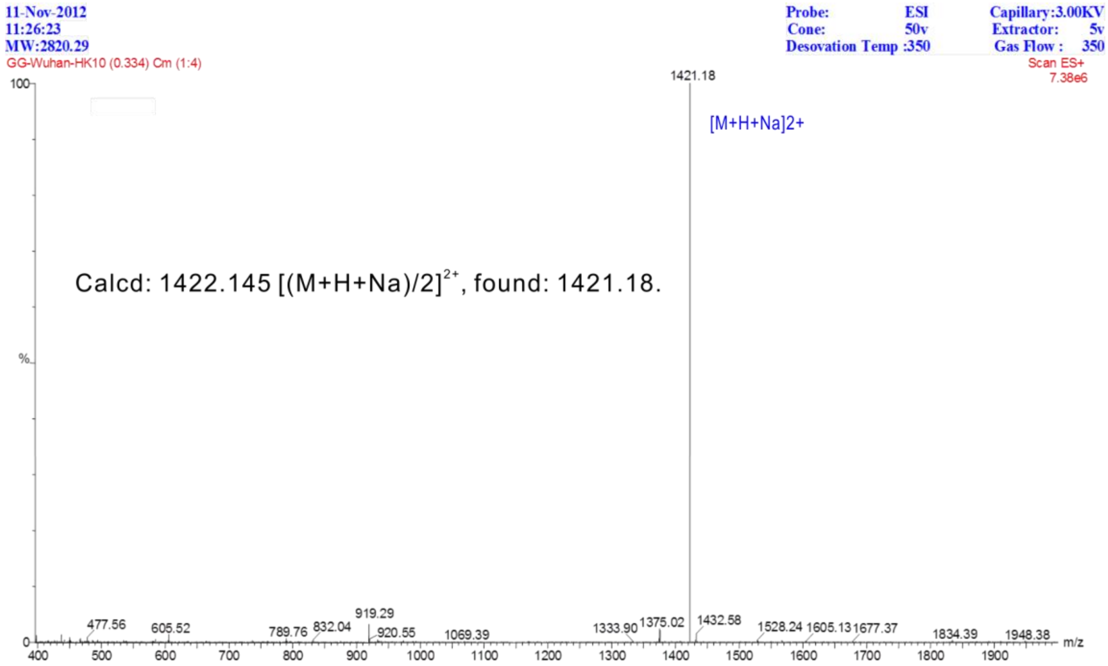

Supplementary Figure 1h. ESI mass spectrum analysis of probe **4**.

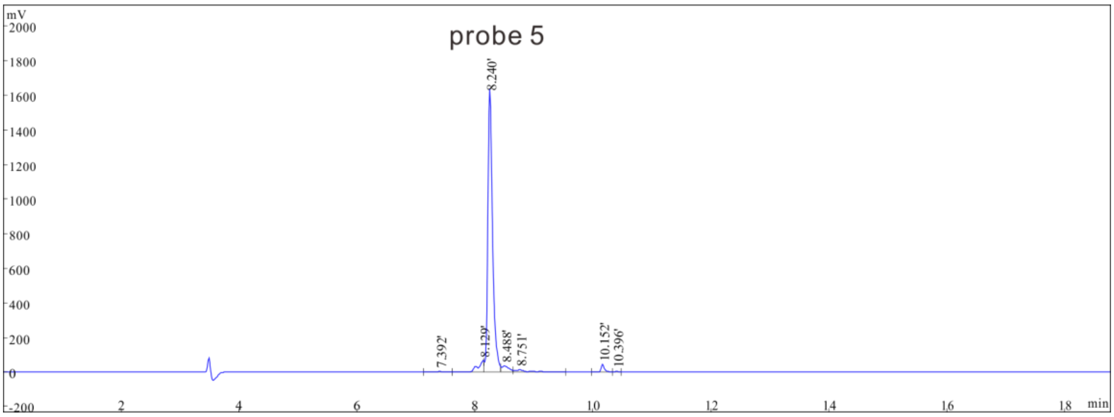

Rank	Time(min)	Conc.(%)	Area
1	7.392	0.4256	45328
2	8.129	5.241	558198
3	8.240	86.86	9251661
4	8.488	3.084	328492
5	8.751	2.611	278075
6	10.152	1.532	163212
7	10.396	0.248	26413
Total		100	10651379

Supplementary Figure 1i. HPLC analysis of probe **5**.

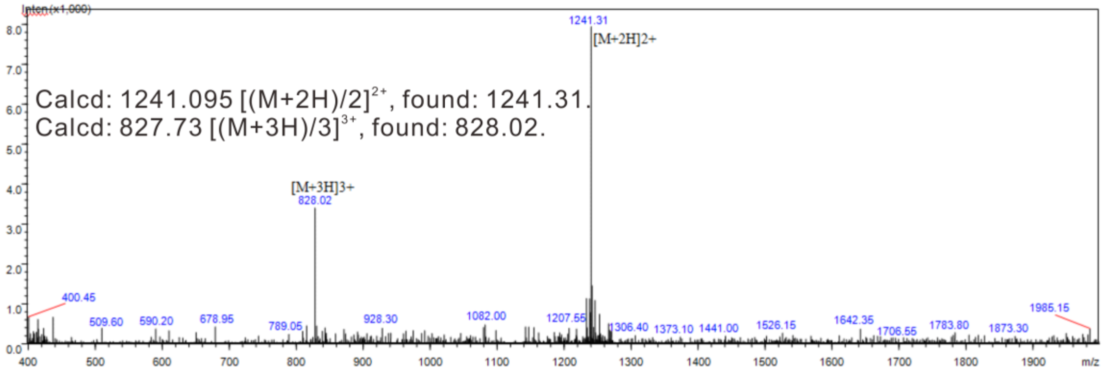

Supplementary Figure 1j. ESI mass spectrum analysis of probe **5**.

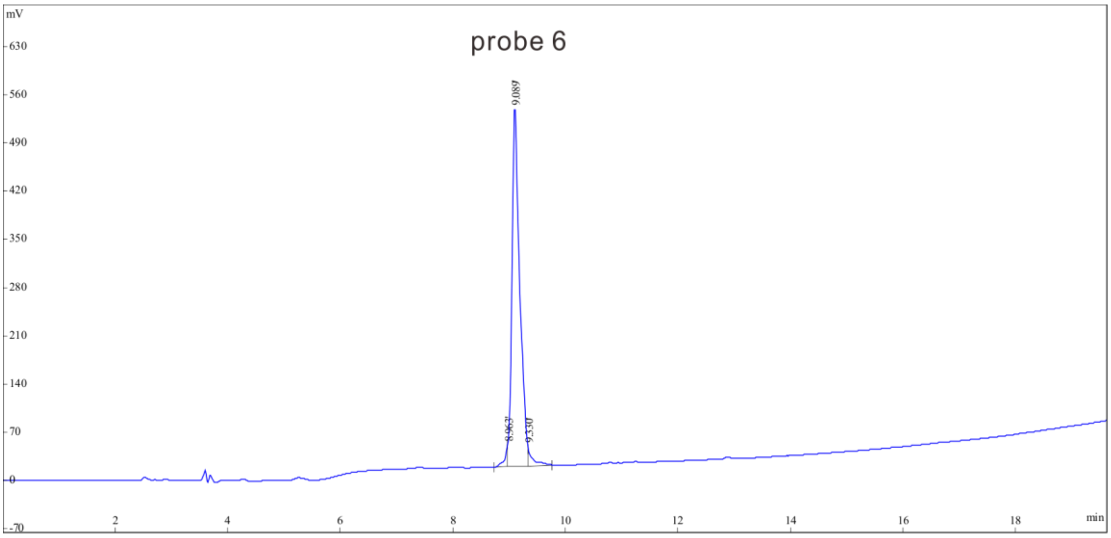

Rank	Time(min)	Conc.(%)	Area
1	8.963	2.123	111132
2	9.089	95.15	4981574
3	9.330	2.73	142935
Total		100	5235641

Supplementary Figure 1k. HPLC analysis of probe **6**.

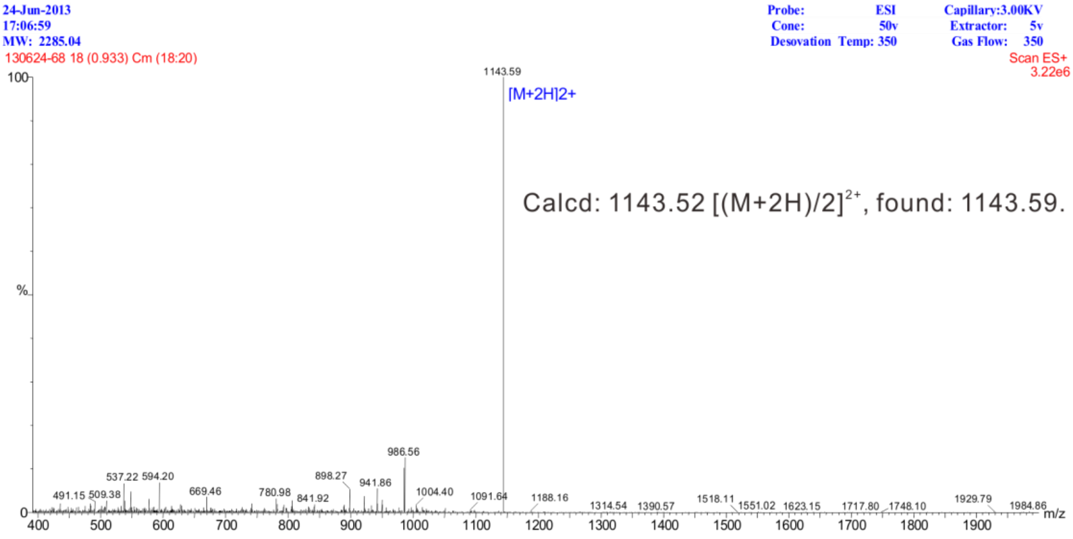

Supplementary Figure 1l. ESI mass spectrum analysis of probe **6**.

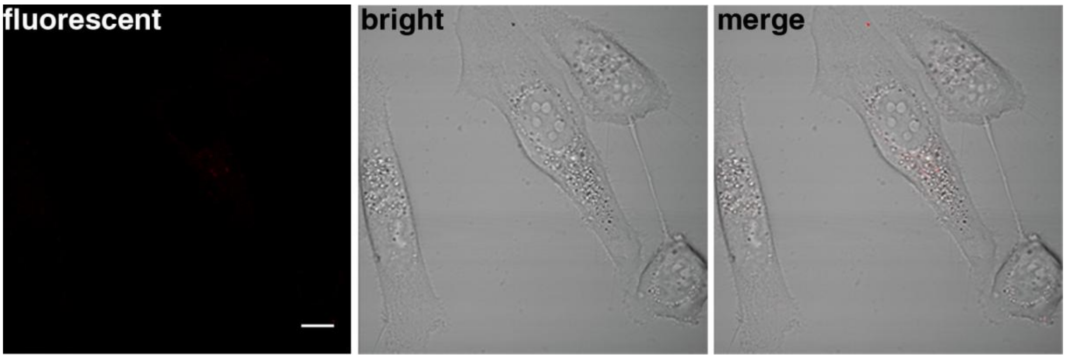

Supplementary Figure 2. The intracellular distribution of probe **4** in live BSC-1 cells.

Confocal microscopy images of live BSC-1 cells after incubation with probe **4** (red, 15 μM) for 30 min. Scale bars: 10 μm.

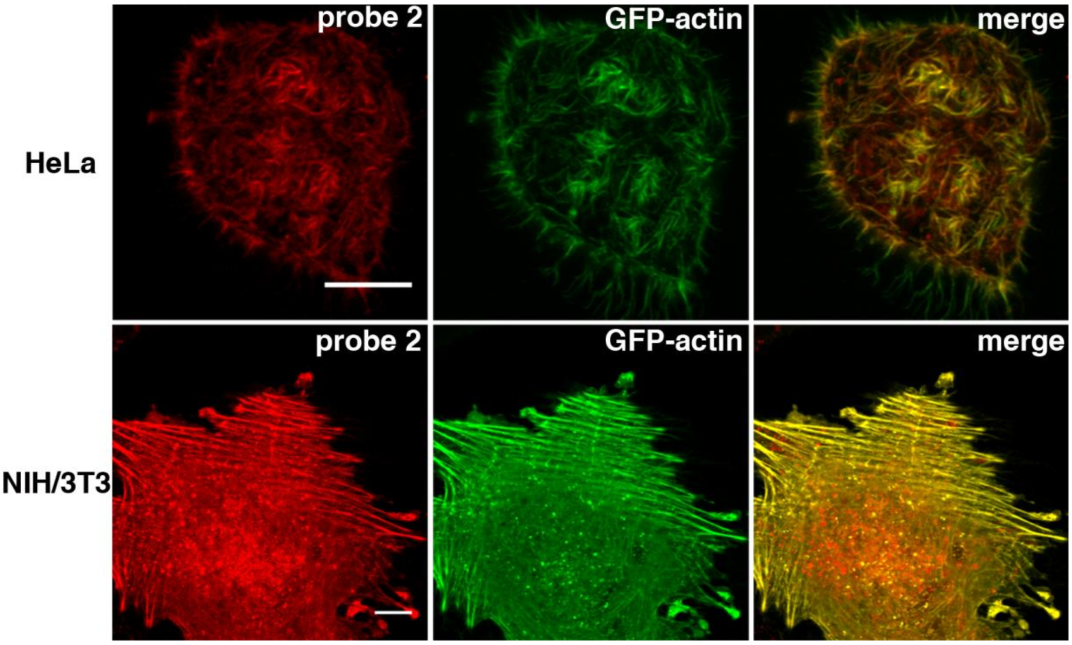

Supplementary Figure 3. Co-localization studies employing GFP-actin as the standard actin marker.

Live HeLa or NIH/3T3 cells transiently transfected with GFP-actin (green) were stained with RhB-labeled probe **2** (red, 15 μM) for 30 min (left) and imaged by confocal microscopy, respectively. Scale bars: 10 μm.

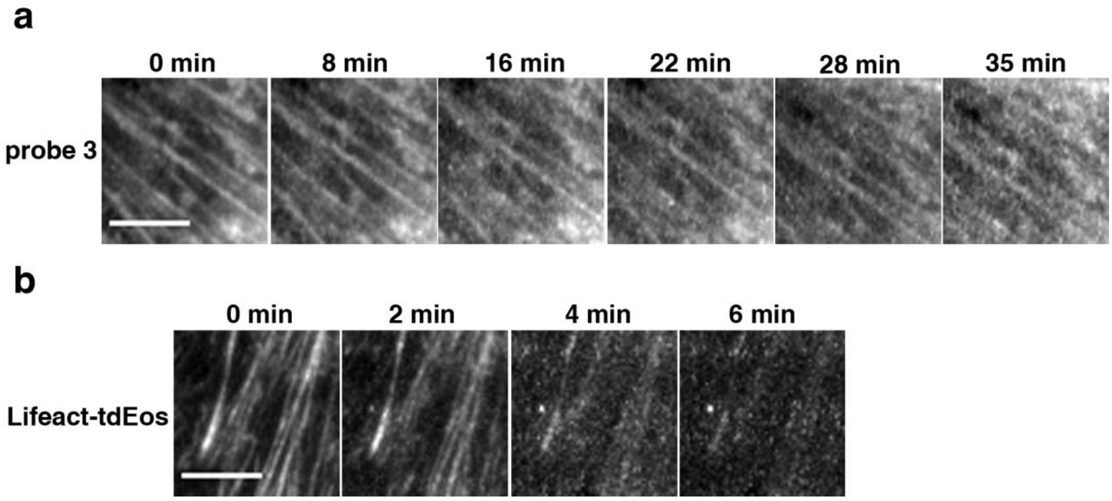

Supplementary Figure 4. Comparison of photobleaching property of PA-RhB-labeled probe **3** and Lifeact-tdEosFP.

a) Live BSC-1 cells were treated with probe **3** (15 μM) for 30 min and then kept being illuminated with a 561 nm laser (0.33 $kWcm^{-2}$) for 35 min. At 0, 8, 16, 22, 28, and 35 min, every 1000 frames were acquired using TIRF microscopy at a frame rate of 100 Hz, respectively, and stacked to give the time-lapse images. Scale bar: 5 μm.

b) Live BSC-1 cells were transfected with Lifeact-tdEosFP and then kept being illuminated with a 561 nm laser (0.165 $kWcm^{-2}$) before Lifeact-tdEosFP was totally photobleached. Every 1000 frames were acquired every 2 min using TIRF microscope at a frame rate of 33 Hz, and stacked to give the time-lapse images. Scale bar: 5 μm.

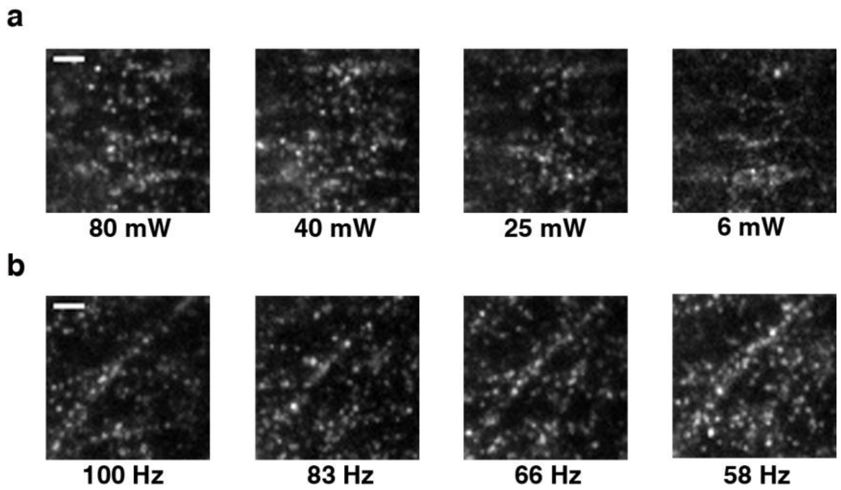

Supplementary Figure 5. The effects of the excitation power and the frame rate on the super-resolution imaging of PA-RhB-labeled probe **3**.

a) Images were acquired at 6, 25, 40 mW or 80 mW, respectively, at 100 Hz. Scale bar: 2 μm.

b) Images were acquired at 100, 83, 66, or 58 Hz, respectively, at 40 mW. Scale bar: 2 μm.

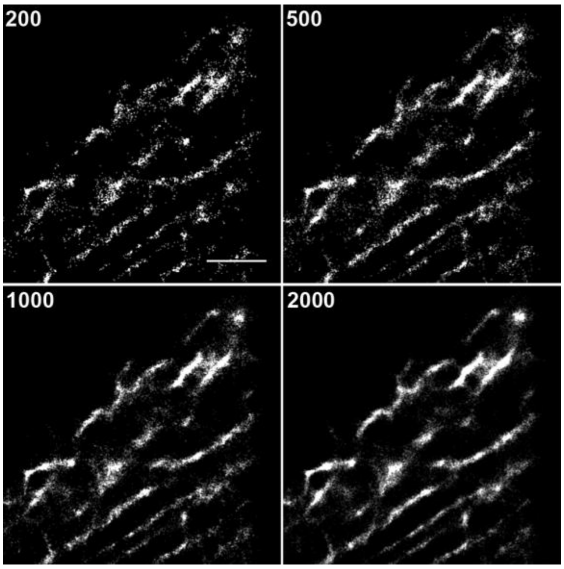

Supplementary Figure 6. Super-resolution images of PA-RhB-labeled probe **3** reconstructed with the PALMER algorithm from 200, 500, 1000, or 2000 frames, respectively.

Frames were acquired at 100 Hz with an excitation power of 0.165 $kWcm^{-2}$ (561 nm). Scale bar: 2 μm.

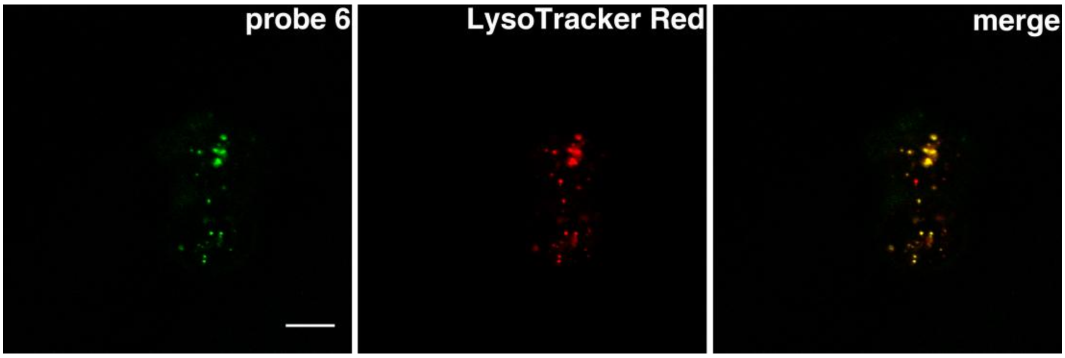

Supplementary Figure 7. Co-localization studies employing LysoTracker Red as the standard lysosome marker.

Live HeLa cells were stained with FAM-labeled probe **6** (15μM) for 30 min and postincubated in DMEM containing 10% FBS for 5 h. Lysosomes were labeled with LysoTracker Red (500 nM) for 30 min before imaging. Scale bar: 10 μm.
